# Serum OPG and RANKL Levels as Risk Factors for the Development of Cardiovascular Calcifications in End-Stage Renal Disease Patients in Hemodialysis

**DOI:** 10.3390/life13020454

**Published:** 2023-02-06

**Authors:** Michalis Spartalis, Efstratios Kasimatis, Eleni Liakou, Erasmia Sampani, Georgios Lioulios, Michalis Christodoulou, Stamatia Stai, Eleni Moysidou, George Efstratiadis, Aikaterini Papagianni

**Affiliations:** Department of Nephrology, Aristotle University of Thessaloniki, “Hippokration” General Hospital, 54642 Thessaloniki, Greece

**Keywords:** osteoprotegerin, RANKL, chronic kidney disease, hemodialysis, cardiovascular calcifications

## Abstract

Cardiovascular calcifications (CVC) are frequently observed in chronic kidney disease (CKD) patients and contribute to their cardiovascular mortality. The aim of the present study was to investigate the impact of osteoprotegerin (OPG)/Receptor Activator of NF-κΒ (RANK)/RANK ligand (RANKL) pathway in the development and evolution of CVCs in hemodialysis patients. In total, 80 hemodialysis patients were assessed for the presence of vascular (abdominal aorta and muscular arteries) calcifications and results were correlated to serum OPG and RANKL levels and the OPG/RANKL ratio. Traditional cardiovascular risk factors and mineral bone disease parameters were also estimated. The presence of VCs was also evaluated 5 years after the initiation of the study, and results were correlated to the initial serum OPG levels. Age, diabetes mellitus, coronary artery disease and OPG levels (*p* < 0.001) were associated with VCs, whereas RANKL levels were not. Multivariate analysis though revealed that only OPG levels were significantly associated with abdominal aorta calcifications (*p* = 0.026), but they were not correlated with the progression of VCs. Serum OPG levels are positively and independently associated with VCs in HD patients, but not with their progression. RANKL levels did not show any associations, whereas further studies are needed to establish the significance of OPG/RANKL ratio.

## 1. Background

Cardiovascular complications are the leading cause of death in patients with chronic kidney disease (CKD), especially in those who are under renal replacement treatment (RRT) [1]. Cardiovascular mortality is 10–20 times higher in patients under hemodialysis (HD) compared to the general population, even after adjustment for age, sex and race.

Cardiovascular calcifications are an almost universal finding in CKD patients and have a major influence in the development of cardiovascular disease. They appear prematurely and evolve quickly, and their extent and type are actual predictors of the subsequent cardiovascular mortality [2,3].

Both traditional and non-traditional risk factors are involved in the pathogenesis of these calcifications. The former ones are frequently observed in patients with CKD, while the non-traditional ones, such as chronic inflammation and oxidative stress, are usually related to the uremic milieu of these patients [4,5].

There are two major types of vascular calcifications, distinguished by their location. The intimal or atherosclerotic type is mostly seen in larger arteries such as the aorta, and it is characterized by calcification of the intimal layer of the vascular wall and gradually leads to the occlusion of the arteries. The second type is the medial artery calcification, otherwise known as Möckenberg sclerosis, which is characterized by an amorphous mineral deposition within the medial layer, which eventually leads to vessel wall stiffness and loss of its elasticity. This type is more prevalent in CKD patients [6,7].

It is now well known that vascular calcification is not just a passive process resulting from calcium and phosphate deposition. It is an active procedure and is highly regulated by complex enzymatic and cellular pathways, resulting in osteogenesis of the vascular wall, where vascular smooth muscle cells (VSMCs), peripheral lymphocytes and macrophages play a central role. During this process, VSMCs, as well as pericytes are differentiated into osteoblast-like cells, thereby regulating the calcification of the vascular wall [5,6,7,8]. Several co-existing parameters, such as diabetes mellitus, older age, oxidative stress and chronic inflammation, together with calcium and phosphate abnormalities, seem to participate in the osteoblastic transformation of VSMCs and the development of cardiovascular calcifications, mainly by dysregulation of the balance between inhibitors/inducers of vascular calcification [5,9,10]. The OPG/RANK/RANKL system is an important part of this active process and plays a significant role in its regulation [9,11,12].

OPG and RANKL are part of the tumor necrosis factor (TNF) superfamily, and they were originally studied as factors involved in the physiology of bone turnover and the immune system [13]. Recent studies, however, revealed that they have a more complicated and remarkable activity, also being the connective link between bone tissue metabolism and vascular wall morphology [11,12,13].

RANKL is a transmembrane protein expressed by T-cells in lymphoid tissue and osteoblasts in areas of bone remodeling, as well as by endothelial and VSMCs in areas of calcifications. After its secretion, RANKL binds to RANK, a transmembrane receptor expressed on osteoclasts and dendritic cells, and regulates the function and survival of these cells, thus increasing bone resorption [13,14]. It also promotes, through activation of the NF-κΒ pathway, the pathological differentiation of healthy VSMCs into osteoblast-like cells, which in turn leads to osteogenesis of the vascular wall [15]. OPG is a dimeric glycoprotein, mainly produced by osteoblasts, immune cells and cardiovascular cells [13,14], and it acts as a decoy receptor to RANKL, thereby preventing the interaction between RANK and RANKL and reducing bone resorption and the calcification process [5,8,9]. Its generation is upregulated by inflammatory modulators and stimuli on VSMCs and endothelial cells, and these elevated OPG levels may exert an anti-calcific effect on the vascular wall and reflect a status of endothelial dysfunction [16,17]. OPG also binds and deactivates the TNF-related apoptosis-inducing ligand (TRAIL), a molecule expressed on many cells, including T-cells and VSMCs and implicated in ectopic mineralization, thereby neutralizing its apoptotic actions [16]. Recent evidence suggests a vasoprotective role for TRAIL, which seems to counteract RANKL’s pro-calcific actions [18], thus implicating another mechanism through which OPG could regulate the calcification process. 

It appears that RANKL and OPG exert the opposite effects on the vascular wall to the ones exerted during bone remodeling. Genetic animal studies have supported this theory, showing that OPG-deficient mice developed severe osteoporosis with the simultaneous appearance of vascular calcifications as well [19,20]. In another study, OPGˉʹ¯/ApoEˉʹˉ VSMCs developed increased calcification after RANKL treatment, whereas OPG⁺^ʹ^⁺/ApoEˉʹˉ cells did not exhibit this result, pointing out a protective role of OPG [21].

Increased levels of serum OPG in the general population, particularly in the elderly and diabetics and patients with ischemic heart disease, are accompanied by increased cardiovascular disease and mortality [22,23,24]. The same association has also been observed in CKD patients and especially in those under RRT, where serum OPG levels are significantly increased [25,26,27], thus generating the question of its true nature and actual action, despite the ones suggested by animal studies. Results concerning the implication and prognostic value of soluble RANKL in the appearance and progression of vascular calcifications are scarce and so far, controversial, whereas the use of OPG/RANKL ratio as a prognostic biomarker of VCs has not been extensively or thoroughly studied with conflicting data to date [12,28].

The aim of the present study was to investigate possible associations of serum OPG and RANKL levels, and their ratio, with the presence and progression of vascular calcifications in CKD patients under HD.

## 2. Methods

### 2.1. Patients and Study Design

Eighty end-stage renal disease patients (ESRD) (42 male—52.5%, and 38 female—47.5%) receiving HD in the Department of Nephrology of Aristotle University, ‘Hippokration’ General Hospital in Thessaloniki, Greece, were enrolled in this study during the first semester of 2015. The study protocol was approved by the Ethics Committee of the School of Medicine, Aristotle University of Thessaloniki, and all protocol procedures were conducted in accordance with the Declaration of Helsinki (2008 Amendment). All patients provided informed written consent prior to enrollment in the study.

Patients included in the study were older than 18 years and had been stable on HD for at least 3 months prior to their enrollment. Exclusion criteria were recent (<3 months) or acute infection, chronic inflammation, active autoimmune disease, previous or active malignancy and finally, treatment with antibiotics, steroids or immunosuppressants for at least 3 months prior to the enrollment.

All information regarding anthropometric and clinical parameters, such as age, sex, weight, dialysis-related parameters, etiology of CKD, co-morbidities and medication at time of enrollment, was gathered by reviewing of their medical records. All patients were receiving dialysis three times per week, for at least 4 h, with a standard bicarbonate containing dialysate.

### 2.2. Laboratory Measurements

Blood samples were collected before a midweek HD session after a 12 h fasting period. Serum levels of glucose, urea, creatinine, total protein, albumin, ALT, AST, total cholesterol, LDL, HDL and triglycerides were determined by routine techniques, using an automated analyzer (Olympus AU560, Hamburg, Germany) at the central laboratory of “Hippokration” General Hospital in Thessaloniki, and they were time averaged for the past 6 months prior to the recruitment to the study. C-Reactive Protein (CRP) was measured by nephelometry, and it was also time averaged for the last 6 months.

Serum calcium, phosphorus, alkaline phosphatase (ALP) and intact parathyroid hormone (iPTH) levels (mineral bone disease markers) were also determined, and they were time averaged for the past 12 months before inclusion in the study. Serum levels of iPTH were measured by radioimmunoassay - RIA (Immunotech, Marseille, France) at the B Internal Medicine Training Clinic laboratory of Aristotle University of Thessaloniki.

Table 1 depicts the demographic characteristics, primary cause of renal failure, comorbid conditions and the dialysis-related parameters of the study population, whereas Table 2 and Table 3 show the participants’ routine laboratory data and their medication, respectively.

### 2.3. Measurement of Osteoprotegerin and sRANKL

Blood samples were drawn from a peripheral vein under fasting conditions in the morning of a midweek routine dialysis session. Serum samples were separated from clotted blood by immediate centrifugation (1500× *g* for 10 min), aliquoted and stored at −70 °C until assay. Serum levels of osteoprotegerin and sRANKL were measured by an enzyme-linked immunosorbent assay (ELISA) using commercially available standard kits (human osteoprotegerin and human sRANKL (total), respectively, BioVendor, Czech Republic). Serum from patients was diluted 1:3 and 1:100, respectively, for the quantitation of osteoprotegerin and sRANKL. The concentrations of these proteins were calculated by reference to standard curves, performed with the corresponding recombinant molecule. All samples were tested in duplicate. The sensitivity of the ELISA system for osteoprotegerin and sRANKL was 0.03 pmol/L and 0.4 pmol/L, respectively.

### 2.4. Clinical Variables

Hypertension was defined as systolic blood pressure ≥ 140 mmHg and/or diastolic blood pressure ≥ 90 mmHg and/or the use of antihypertensive drugs. Diabetes mellitus (DM) was considered present if the patient was on antihyperglycemic medication or had fasting glucose levels > 126mg/dL. Coronary artery disease (CAD) was defined either as at least one documented episode of angina pectoris or a history of myocardial infraction or a coronary stenosis >75%, evidenced by coronary angiography. Cardiovascular disease (CVD) was considered if a patient had a history of CAD and/or atrial fibrillation (AF) and/or peripheral artery disease (PAD) and/or cerebrovascular accident (CVA). Body mass index (BMI) was defined as the post HD body weight (kilograms) divided by height squared (meters).

### 2.5. Vascular Calcifications

Vascular calcifications (VCs) were assessed using the Adragao and Kauppila scores, whose methods of estimation and correlation with VCs and cardiovascular outcome has been previously prescribed [29,30]. Adragao score was evaluated with the use of pelvis and hands X-rays which revealed calcifications of the iliac, femoral, radial and digital arteries (muscular arteries calcifications (MACs), and Kauppila score with the use of lateral lumbar spine calcifications, which showed abdominal aorta calcifications (AACs). The assessment of the calcifications was performed by a radiologist blinded to the patients’ clinical and laboratory characteristics. According to their radiological scores, the patients were divided into the following categories: (1) Adragao1: patients with Adragao score 0–2; (2) Adragao2: patients with Adragao score 3–8; (3) Kauppila1: patients with Kauppila score 0–4; (4) Kauppila2: patients with Kauppila score 5–24. Patients with either Adragao2 or Kauppila2 scores were considered as having severe VCs and a higher cardiovascular risk; therefore, based on the above measurements, we further categorized our patients into two groups. The first one was the low calcification score and low cardiovascular risk group, which consisted of patients with both Adragao1 and Kauppila1 scores, whereas the second group—high calcification score and high cardiovascular risk—consisted of patients with at least one high radiological score, either Adragao 3–8 or/and Kauppila 5–24.

Five years after the initial enrollment in the study, 47 patients had new pelvis, hand and lateral lumbar spine X-rays which were evaluated for the severity of VCs, and patients were again divided into the above-mentioned groups. Cox regression analysis was then performed to evaluate the association of the clinical and biochemical biomarkers that the patients had upon enrolment, with the progression of their VCs.

### 2.6. Statistical Analysis

Statistical analysis was performed using the IBM Statistical Package for Social Sciences (SPSS) Statistics v26 for windows. The Shapiro–Wilk or the Kolmogorov–Smirnov tests were applied to examine the normality of the distribution for continuous variables. Data from normally distributed and non-normally distributed variables were expressed as Mean ± Standard Deviation and Median and Interquartile Range, respectively. Similarly, differences between groups were estimated using Student’s *t* test for independent samples or Mann–Whitney U test, respectively. Pearson’s and Spearman’s coefficients were used for the correlation between normally and non-normally distributed variables. Odds Ratio (OR) and Receiver Operating Characteristics (ROC) curves were applied to estimate the incidence of serum OPG levels in the presence of vascular calcifications. Multivariate analysis was performed to evaluate serum OPG levels and other independent parameters contributing to the presence of vascular calcifications. Finally, Cox regression analysis was performed to estimate the contribution of OPG serum levels to the progression of vascular calcifications 5 years after the enrollment of the patients. Values of *p* < 0.05 (two-tailed) were considered statistically significant for all comparisons.

## 3. Results

Eighty patients, M/F 52.5%/47.5%, mean age of 57.2 ± 15 years, on standard hemodialysis treatment for a mean time of 64 ± 58 months, were included in the study.

The mean value of OPG was 47.6 ± 23.8 pmol/L, of RANKL 455.34 ± 1002.02 pmol/L and of OPG/RANKL ratio 0.40 ± 0.44.

### 3.1. The Severity of Cardiovascular Calcifications and Correlations with Clinical and Laboratory Parameters

Based on plain X-rays, 29/80 (36.3%) patients had a high Adragao score (3–8), and 39/80 (48.8%) had a Kaupilla score of 5–24. Thirty-five patients (43.7%) were considered to have a low calcification score and low cardiovascular risk, while 45 patients (56.3%) had either an Adragao score of 3–8 or/and a Kauppila score of 5–24 and were considered as having a high calcification score and a high cardiovascular risk. 

Correlations between the severity of vascular calcifications and clinical and biochemical parameters are shown in Table 4.

Age, presence of DM, CAD and CVD were associated with both categories of VCs and a higher calcification score. The use of statins and anti-platelet agents was also associated with the existence of VCs, which most probably reflects the cardiovascular risk of these patients. Serum phosphate levels and elevated BMI had positive correlations with MACs and a high calcification score, whereas hypertension was correlated with AACs. On the other hand, gender, smoking, dyslipidemia, serum albumin, ALP, CRP and iPTH levels did not show statistically important associations with the presence of any VCs in the study population.

Serum OPG levels were strongly and positively associated with both categories of VCs (*p* < 0.001) and a high calcification score (Table 5). OPG/RANKL ratio was positively associated with AACs and calcification score, whereas RANKL levels were not associated with the presence of VCs. 

**Figure 1:**
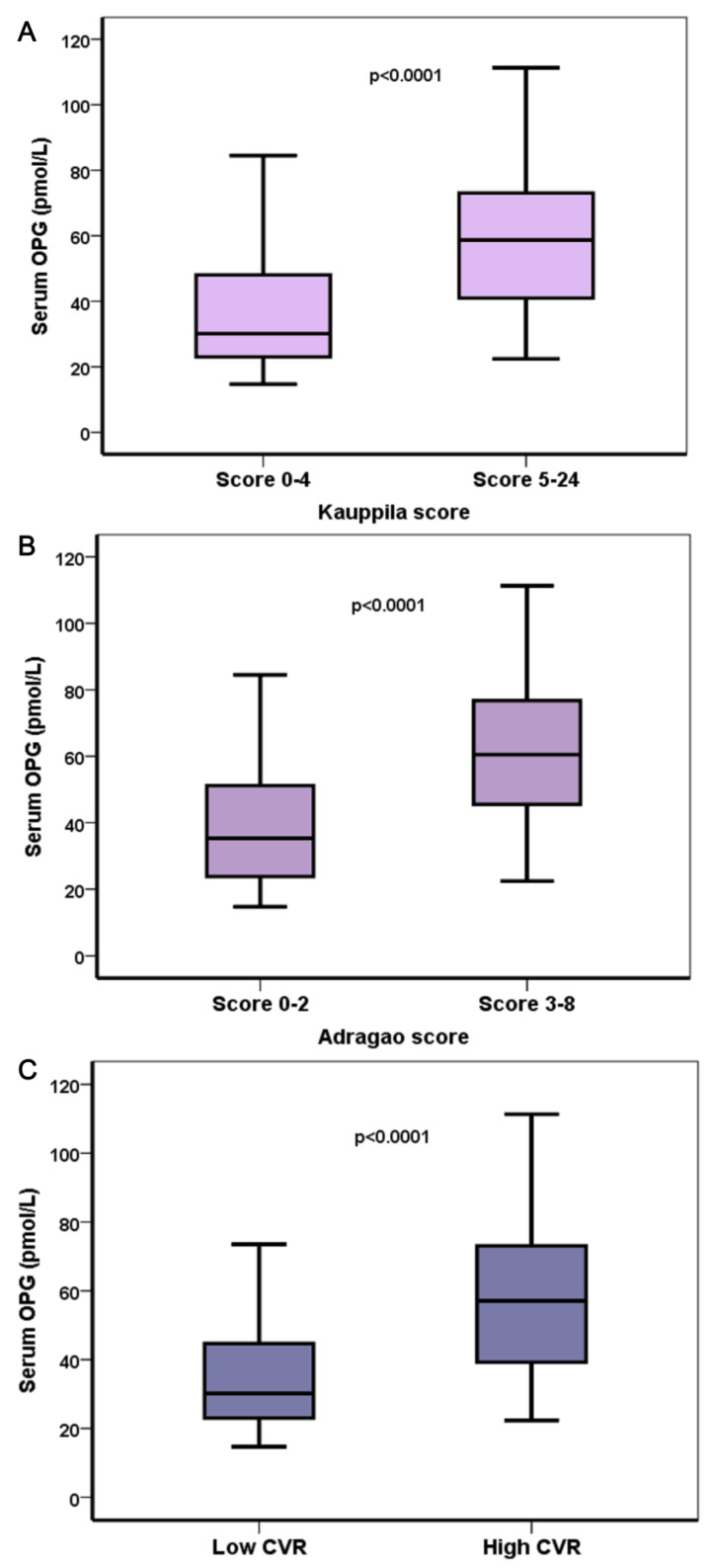
Differences in serum OPG levels according to Kauppila score (**A**), Adragao score (**B**) and according to cardiovascular risk (**C**). Elevated serum OPG levels were strongly and positively associated with increased Adragao and Kauppila cores and a high cardiovascular risk.

**Figure 2:**
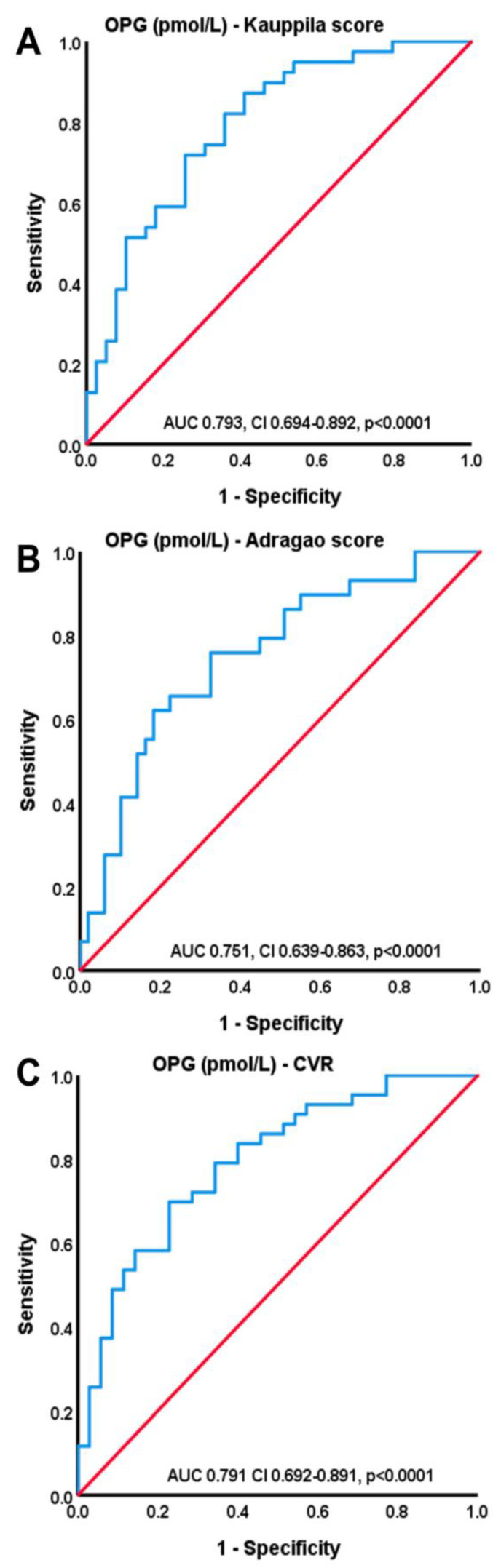
ROC curves of the OPG serum levels and Kauppila score (**A**), Adragao score (**B**) and according to cardiovascular risk (**C**).

Figure 1 shows differences in OPG levels according to the severity of cardiovascular risk and VCs, whereas the ROC curves depicted in Figure 2 show the importance of OPG serum levels in the severity of VCs as this was estimated by the Kauppila score (AUC 0.793, CI 0.694–0.892, *p* < 0.001), Adragao score (AUC 0.751, CI 0.639–0.863, *p* < 0.001) and cardiovascular risk (AUC 0.791, CI 0.692–0.891, *p* < 0.001).

### 3.2. Multivariate Analysis

In a logistic regression modeled analysis, age, DM, the presence of CVD, hypertension and serum OPG levels were evaluated as independent variables, predicting the presence of AACs. A second modeled analysis was also performed for the presence of MACs, where the independent variables examined were age, DM, CVD, BMI and serum OPG levels.

Since serum RANKL levels and OPG/RANKL ratio did not have statistically significant correlations with VCs at the univariate analysis, we only used serum OPG levels for the multiple logistic regression analysis.

The results of both models are depicted in Table 6. Serum OPG was the only variable that retained its statistical significance at the end of the regression analysis for the presence of AACs (*p* = 0.026), whereas age failed to do so. Concerning muscular arteries calcifications, BMI was actually the only variable that was positively correlated with them after the multivariate analysis (*p* = 0.020).

### 3.3. Correlations of Clinical Parameters and Initial Serum OPG Levels with the Progression of VCs

Five years after the initial enrolment in the study, 47 patients had new X-rays done. Out of the remaining 33 patients, 22 patients died before the completion of the 5-year follow-up, 1 patient had a transplantation, and 10 patients were lost to follow-up, mostly because of transfer to other dialysis centers. In total, 8 out of the 47 patients (17%) showed a significant progression of AACs and were categorized in the Kauppila2 group, whereas upon enrollment they were in Kauppila1 group. Concerning MACs, this percentage was lower with 5/47 patients (10.6%) changing their group categorization to Adragao2. The change in group categorization—and hence the progression of VCs—was used as a variable in the Cox regression analysis model. Age, serum OPG levels and hypertension were included as independent variables predicting the progression of AACs, whereas BMI was used instead of hypertension in the regression analysis of MACs. 

Besides the change in group categorization, we also used the actual increase in VCs as a variable, since it also represents their progression. Patients who presented a doubling—at the least—of their Kauppila score and/or also patients who increased their Adragao score by 1.5 were included in another Cox regression analysis model, using the above-mentioned independent variables. In total, 9/47 patients had a doubling of their Kauppila score (19.1%), and 6/47 increased their Adragao score by 1.5 (12.8%).

Despite the strong association that serum OPG levels had with the presence of VCs upon enrollment, they were not associated with their progression. In contrast, age showed a positive association with the progression of AACs (*p* = 0.022 in the group change, and *p* = 0.024 in the doubling of the Kauppila score), whereas BMI was positively associated (*p* = 0.001) with the change in Adragao group.

## 4. Discussion

The association of OPG, RANKL and their ratio with cardiovascular calcifications in ESRD patients has been investigated in previous studies but the exact nature of this association remains unclear, as well as its prognostic significance.

The aim of this study was to verify whether these molecules are associated with the presence and progression of vascular calcifications, as well as with the cardiovascular risk in hemodialysis patients.

Evidence acquired over the years suggests that the RANK/RANKL/OPG pathway has a connective role between bone remodeling and vascular calcification and simultaneously acts on osteoblasts and osteoclasts as well as on endothelial cells and VSMCs [12,31,32]. This connecting role is further supported by the fact that OPG is constitutively expressed on the normal vascular wall—contributing most probably to the maintenance of its morphology—and on the surface of endothelial cells and VSMCs. It is rapidly secreted in response to inflammatory stimuli, inhibits the osteoclast activity, acting as a decoy receptor for RANKL, and at the same time, it promotes endothelial cell survival through its anti-apoptotic actions (TRAIL system) [12,31]. In contrast, RANKL and RANK are mostly undetectable on the normal vascular wall, but their expression is significantly increased in calcified areas [16]. Immunohistochemistry performed on calcified areas of the arterial wall of CKD patients revealed that the calcification process is strongly regulated by immunological factors and that the degree of vascular calcification was positively correlated with the intensity of OPG expression, whereas intima media thickness was associated with the degree of RANKL expression [33].

Several studies suggest an association between bone mineral metabolism and vascular calcifications in CKD patients and an impact of bone turnover on their development [34]. There is also evidence of the reduced progression of VCs after the improvement in bone status [35]. In HD patients, additional inhibition of bone resorption by elevated serum OPG levels could result in the inability to accumulate calcium and phosphorus to the bone and metastatic calcification of the vascular tree. Since OPG and RANKL have opposing effects on bone resorption, the OPG/RANKL ratio could be used as a marker of bone turnover—with a high OPG/RANKL value representing a state of low bone turnover—and as a biomarker for the presence of cardiovascular calcifications too.

Age, DM and the presence of CAD and CVD were all positively associated with VCs in our study. Hypertension was correlated with AACs, whereas serum phosphate levels and an elevated BMI were positively associated with MACs. These results are in accordance with previous studies, showing the implication of traditional risk factors in the appearance of cardiovascular calcifications [36,37]. Univariate analysis also revealed a strong and positive association of serum OPG levels with VCs, in line with results published from other studies and suggesting its possible role in the pathogenesis and appearance of VCs [38,39]. 

At the end of the multivariate analysis, serum OPG levels were the only variable that was independently associated with the presence of AACs. Previous studies carried out on pre-dialysis CKD patients have also indicated serum OPG concentration as an independent predictor of VCs, one of them also pointing out a cut-off value of plasma OPG level as a prognostic biomarker for the presence of coronary artery calcifications (CACs) [38,40]. To our knowledge, our study is the first to reveal such a strong and independent association of OPG with AACs in hemodialysis patients, even after adjustment to traditional risk factors including age, thus highlighting the prognostic importance of OPG in younger patients in dialysis. A study by Avila et al. also revealed OPG to be the strongest risk factor associated with arterial calcifications; however, this was performed on peritoneal dialysis patients [41].

The results concerning the presence of MACs were different. After the multivariate analysis, BMI was the only variable that kept its statistical and positive significance. This result comes in accordance with other studies which have associated BMI with VCs, performed both on the general population [42,43] and CKD patients [36]. This is the first study though to reveal BMI as a strong and independent predictor of MACs in hemodialysis patients, affirming its implication and importance. An elevated BMI reflects an excess of adipose tissue, which in turn acts as an inflammatory stimulus that leads to a cascade of actions and eventually vessel atheromatosis, calcification and peripheral artery disease [44].

Our study enhances the assumption that traditional risk factors alone cannot explain the increased incidence of VCs and cardiovascular disease in HD patients, and uremic-related factors are also implicated. OPG remained a strong and independent factor and seems to act as a very potent prognostic biomarker, compared to other traditional risk factors such as age and DM. Its exact function though is still not known and the exact mechanism behind this association is still controversial. Increased OPG levels might either be a result of the vascular damage and the endothelial malfunction observed in CKD patients, or they might have a crucial role in the calcification process itself, or they rise as a compensatory protective mechanism, to counteract the appearance and the progression of vascular calcifications.

Results of the Cox regression analysis that we performed suggest that serum OPG levels are not associated with the progression of vascular calcifications, even though they were associated with their presence at the beginning of the study. Previous studies have so far given conflicting information regarding the association of serum OPG with the progression of VCs. Kurnatowska et al. have supported the use of plasma OPG as a marker of the progression of calcification in HD patients [45], and in a study by Ozkok et al., baseline OPG levels were correlated with the progression of CACs at the end of the one-year follow-up. However, after the linear regression analysis, only baseline CAC score and the difference in OPG levels were associated with the progression of CACs and not baseline serum OPG levels [46]. Animal studies have also supported the role of OPG as an inhibitor and a marker of calcification and not as a mediator of atheromatosis [39], and the results obtained by Moldovan et al. support the increase in serum OPG levels as a response and defense to vascular injury [47]. Another study performed on pre-dialysis and hemodialyzed patients did not find any association between OPG levels and the progression of VCs after a 4-year follow-up [48]. These studies support the result obtained from our regression analysis, suggesting that increased serum OPG levels reflect a status of endothelial damage and a compensatory mechanism to protect the vascular wall. On the other hand, age and BMI were both associated with the progression of vascular calcifications in our study. This points to the fact that even though the pathogenesis of VCs is complex in CKD patients, the importance of older age and obesity remains unquestionable.

As far as soluble RANKL is concerned, we did not find any significant correlations at all neither with the clinical and laboratory parameters of the study population nor with the presence of vascular calcifications. Despite the growing evidence showing the implication of RANKL in the osteoblastic differentiation of VSMCs [49], the current data concerning the relationship of sRANKL and VCs are controversial. Ozkok et al. showed a significant negative correlation between sRANKL values and CACs at baseline and at one-year follow-up in HD patients [46] and another study by Wei et al. demonstrated a positive association between cardiovascular events and low serum RANKL levels in HD patients [50]. However, other studies performed in CKD patients and general population did not show any association of soluble RANKL with calcification [51,52]. Apparently, RANKL plays an important role in the pathogenesis of VCs, but the exact nature and the significance of the relationship between sRANKL levels and VCs still remains inconclusive. 

The results concerning the OPG/RANKL ratio are more promising. It was positively correlated with the presence of AACs and a high calcification score. This result is also supported by Ozkok et al. [43], where OPG/RANKL ratio values were higher both at baseline and after 1 year of follow-up, in the group of patients who showed a progression of CACs, compared to the non-progressive group. Its use though as a prognostic biomarker of VCs has not been evaluated, and since in our study sRANKL did not show any associations with VCs or any of the other variables, we could attribute the results regarding the association of OPG/RANKL with VCs to the presence of OPG in the equation and not to the ratio itself. Further studies should be carried out to establish the use of the OPG/RANKL ratio as an indicator and regulator of bone turnover and as a prognostic biomarker for the appearance of cardiovascular calcifications. 

Our study definitely has some limitations. It was a cross sectional study, and the number of patients was relatively small, which did not let us include more variables in our modeled analysis. We should also mention that only 11.3% of our study population had DM, whereas diabetic patients usually comprise about 30–40% of CKD patients on dialysis, and DM is known to be associated with CVCs. Our study population was also relatively young, with a mean age of 57 years, which may be the reason why age did not retain its significance in the multivariate analysis. However, this does not eliminate the importance of OPG and its association with VCs. It also emphasizes the fact that in younger patients on dialysis, OPG might be a stronger and more important biomarker than age and other clinical parameters concerning the presence of VCs and could help detect patients with increased cardiovascular risk. Another limitation is the fact that only 47 patients had new X-rays done at the 5-year follow-up, which diminishes the strength of the Cox regression analysis. It would also be better if instead of BMI, we used actual visceral adiposity measurements to evaluate the association of adipose tissue with VCs, since BMI is not always representative of it. Visceral adiposity measurements are more complex though and not always available, so simple BMI measurements can still be used as another tool and lead to relatively safe results. 

## 5. Conclusions

The OPG/RANKL/RANK pathway is considered to play a significant role in the emergence of vascular calcifications in CKD patients. Even though the results are still conflicting, our study reveals that serum OPG levels are strongly associated with vascular calcifications and can be safely used as an independent prognostic marker for them, and this association seems to be of particular interest to younger patients on hemodialysis. Our study also showed that OPG levels are not related to the progression of VCs. Its exact role in the process of vascular calcification is still obscure, as its multiple and conflicting effects remain unspecified and will need further elucidation. On the other hand, RANKL levels do not seem to be associated with the presence of CVCs, whereas more information is needed concerning the use of OPG/RANKL ratio as a prognostic biomarker.

## Figures and Tables

**Table 1:** General demographic and clinical characteristics of the study population.

Age (yrs) (M ± SD) *	57.2 ± 15.4
Gender (N, %) *	
Male	42 (52.5%)
Female	38 (47.5%)
BMI (Kg/m^2^) (M ± SD)	26.1 ± 4.9
Dialysis parameters (M ± SD)	
Dialysis session (min)	245 ± 13.5
Time on dialysis (months)	64.3 ± 58
Primary cause of ESRD (N, %)	
Primary Glomerulonephritis	23 (28.7%)
Polycystic Kidney disease	11 (13.7%)
Diabetic nephropathy	7 (8.8%)
Chronic Interstitial nephritis	6 (7.5%)
Obstructive uropathy	4 (5%)
Alport’s syndrome	4 (5%)
Other reasons	25 (31.3%)
Comorbid conditions (N, %)	
Smoking	21 (26.3%)
Hypertension	53 (66.3%)
Diabetes Mellitus (DM)	9 (11.3%)
Coronary artery disease (CAD)	11 (14.3%)
Atrial fibrillation (AF)	16 (20.8%)

* Values are expressed as the mean ± standard deviation (SD) or as number (percentage) of patients.

**Table 2:** Laboratory characteristics of the study population.

Characteristics, M± SD	
Serum urea (mg/dL)	136 ± 22.5
Serum creatinine (mg/dL)	9 ± 2.6
Hgb (g/dL)	11.5 ± 1.15
Serum Calcium (mg/dL)	9.1 ± 0.54
Serum Phosphorus (mg/dL)	4.97 ± 0.98
Serum Glucose (mg/dL)	94 ± 19.4
Serum Albumin (g/dL)	4.6 ± 4.9
Serum ALP (U/L)	105 ± 77.5
Serum AST (IU/L)	14.3 ± 8.3
Serum ALT (IU/L)	13.8 ± 11.3
Serum Triglyceride (mg/dL)	152 ± 78.6
Serum Cholesterol (mg/dL)	157 ± 33
Serum HDL (mg/dL)	44.7 ± 15.1
Serum LDL (mg/dL)	84.8 ± 29
Serum CRP (mg/L)	6.7 ± 8.5
Serum iPTH (pg/mL)	396 ± 296
Kt/V	1.47 ± 0.27

**Table 3:** Medication provided to the study population.

RAAS inhibitors	14 (17.5%) *
b-blockers	37 (48.7%)
CCBs	24 (31.6%)
Statins	29 (36.3%)
Anti-PLT agents	42 (52.5%)
Sevelamer	62 (77.5%)
Lanthanum Carbonate	27 (33.8%)
Calcium Carbonate	10 (12.5%)
VDR agonists	47 (58.8%)
Cinacalcet	27 (33.8%)
CCPBs	17 (21.3%)
NCCPBs	71 (88.8%)

* Values are expressed as number (percentage) of patients receiving the medication. RAAS—renin angiotensine aldosterone system; CCBs—calcium channel blockers; Anti-PLT—antiplatelet agents (aspirin, clopidogrel); VDR—vitamin D receptor; CCPBs—calcium-containing phosphate binders; NCCPBs—non-calcium-containing phosphate binders.

**Table 4:** Associations of vascular calcifications with clinical and laboratory parameters.

	Kauppila Group	Adragao Group	Calcscore
Age	*p* < 0.001	*p* < 0.001	*p* < 0.001
Hypertension	*p* = 0.006	NS	*p* = 0.013
DM	*p* =0.016	*p* < 0.001	*p* = 0.005
CAD	*p* = 0.004	*p* = 0.001	*p* = 0.001
CVD	*p* = 0.001	*p* = 0.001	*p* = 0.001
Time on dialysis	NS	*p* = 0.036	NS
Dialyzer	NS	NS	NS
Use of statins	*p* = 0.004	*p* = 0.030	*p* = 0.002
Use of anti-PLTs	*p* = 0.007	*p* = 0.040	*p* = 0.002
Use of ARBs	NS	NS	NS
Use of b-blockers	*p* = 0.039	*p* = 0.038	NS
Use of NCCPBs	NS	NS	NS
Serum Calcium (mg/dL)	NS	NS	NS
Serum Phosphorus (mg/dL)	NS	*p* = 0.028	*p* = 0.045
Serum iPTH (pg/mL)	NS	NS	NS
Serum urea (mg/dL)	NS	NS	NS
Serum creatinine (mg/dL)	NS	NS	NS
Serum glucose (mg/dL)	NS	NS	NS
CRP (mg/L)	NS	NS	NS
BMI (kg/m²)	NS	*p* = 0.001	*p* = 0.036

Calcscore—calcification score, DM—diabetes mellitus, CAD—coronary artery disease, CVD—cardiovascular disease, ARBs—angiotensin II receptor blockers, NCCPBs—non calcium containing phosphate binders, CRP—C reactive protein, BMI—body mass index; *p* < 0.05 was considered statistically significant.

**Table 5:** OPG, RANKL and OPG/RANKL correlations with vascular calcifications.

	Univariate Analysis		
	OPG	RANKL	OPG/RANKL
Kauppila gr	*p* < 0.001	NS	*p* = 0.022
Adragao gr	*p* < 0.001	NS	NS
CalcScore	*p* < 0.001	NS	*p* = 0.014

*p* < 0.05 was considered statistically significant.

**Table 6:** Multiple logistic regression analysis for the presence of vascular calcifications.

	Kauppila gr	Adragao gr
OPG levels	*p* = 0.020	NS
Age	NS	NS
CVD	NS	NS
Diabetes mellitus	NS	NS
Hypertension	NS	Not included
BMI	Not included	*p* = 0.026

CVD—cardiovascular disease; BMI—body mass index; OPG—osteoprotegerin. *p* < 0.05 was considered statistically significant.

## Data Availability

The data presented in this study are available on request from the corresponding author.

## Abbreviations

AACs—abdominal aorta calcifications; AUC—area under the curve; BMI—body mass index; CACs—coronary artery calcifications; CAD—coronary artery disease; CI—confidence interval; CKD—chronic kidney disease; CVCs—cardiovascular calcifications; CVD—cardiovascular disease; DM—diabetes mellitus; ELISA—enzyme-linked immunosorbent assay; ESRD—end-stage renal disease; MACs—muscular arteries calcifications; OPG—osteoprotegerin; RANKL—receptor activator of nuclear factor κΒ ligand; RRT—renal replacement therapy; TNF—tumor necrosis factor; TRAIL—tumor-necrosis-factor-related apoptosis-inducing ligand; VCs—vascular calcifications; VSMCs—vascular smooth muscle cells.
